# Simplified PADUA renal nephrometry system, an imaging features scoring system, predicts perioperative outcomes in partial nephrectomy: a meta-analysis

**DOI:** 10.3389/fonc.2026.1684584

**Published:** 2026-05-20

**Authors:** Zhiqiang Zeng, Xionglin Hu, Tao Zhou, Tao Li, Huan Zhao, Peng Ji, Yang He, Yubo Zhou, Wubin Chen, Wangbing Chen, Lunhong Zou

**Affiliations:** 1 Department of Urology, Santai People’s Hospital (Santai Hospital Affiliated to North Sichuan Medical College), Mianyang, Sichuan, China; 2 North Sichuan Medical College, Nanchong, Sichuan, China

**Keywords:** meta-analysis, nephrometry system, partial nephrectomy (PN), renal cancer, simplified PADUA renal (SPARE) nephrometry system

## Abstract

**Objective:**

Does the simplified PADUA renal (SPARE) nephrometry system predict perioperative outcomes after partial nephrectomy (PN)?

**Methods:**

This systematic review and meta-analysis were conducted in accordance with the PRISMA statement. A systematic search of the literature published before August 1, 2024, was conducted using PubMed, Embase, Cochrane, and Web of Science libraries. We included all articles patients with SPARE score for PN.

**Results:**

A total of 1,831 patients from six studies meeting the criteria were included. Higher SPARE scores were significantly associated with several perioperative outcomes after partial nephrectomy and may serve as a practical tool for preoperative tumor complexity assessment and perioperative risk stratification, particularly in the context of robot-assisted partial nephrectomy given the composition of current evidence.

**Conclusions:**

The SPARE score was significantly associated with several perioperative outcomes after partial nephrectomy and may serve as a practical tool for preoperative tumor complexity assessment and perioperative risk stratification.

**Systematic review registration:**

https://www.crd.york.ac.uk/PROSPERO/view/CRD42024578763, identifier CRD42024578763.

## 1 Introduction

Renal cancer represents a substantial and increasing global health burden. Recent global epidemiological data based on Global Cancer Observatory estimates reported 434,840 new renal cancer cases and 155,953 deaths worldwide in 2022, with projected increases to 745,791 new cases and 304,861 deaths by 2050 (1–3). Its development is considered multifactorial, involving both genetic predisposition and environmental or metabolic risk factors (4, 5). Major non-modifiable risk factors include sex, geography, ethnicity/ancestry, and family history, while major modifiable risk factors include obesity, diabetes, hypertension, chronic kidney disease, smoking, and environmental exposure (6). For localized renal tumors, surgery is still favored as the primary treatment (7, 8). The primary surgical options for renal malignancy are partial nephrectomy (PN) and radical nephrectomy (RN), both of which aim to achieve effective oncologic control while preserving renal function and considering the patient’s overall health status. For T1 renal tumors, as well as selected higher-stage tumors, PN is the preferred treatment (9–12). A multicenter study evaluated the outcomes of 1,800 patients who underwent either open partial nephrectomy (OPN) or robot-assisted partial nephrectomy (RAPN). Compared with the OPN group, the RAPN group had a shorter hospital stay, lower rates of overall and major complications, and a reduced need for blood transfusion (13).

PN is a complex surgical procedure that involves complete tumor excision followed by renal reconstruction. Preoperative assessment of tumor anatomy is essential. Knowledge of tumor characteristics, including size, location, and vascular supply, facilitates surgical planning and helps predict potential postoperative complications (14, 15).

Several nephrometry scoring systems have been proposed to standardize the assessment of renal masses and guide surgical decision-making. These systems mainly include the R.E.N.A.L. and PADUA scoring systems, which are based on anatomical factors; the C-index, which reflects the geometric relationship between the tumor and the kidney; and the Mayo adhesive probability (MAP) score, which assesses perirenal fat (16–19).

Early nephrometry systems had several limitations, including suboptimal interobserver reproducibility and incomplete assessment of relevant anatomical features (20, 21). To simplify the scoring process, improve prediction of perioperative outcomes, enhance reproducibility, and increase the accuracy of the original PADUA system, Ficarra et al. proposed the Simplified PADUA Renal (SPARE) scoring system (22). The SPARE scoring system includes four components: (1) rim location, (2) renal sinus involvement, (3) exophytic rate, and (4) tumor size.

Therefore, we conducted this systematic review and meta-analysis to evaluate the association between the SPARE score and perioperative outcomes after PN and to clarify its potential role in preoperative surgical complexity stratification.

## 2 Methods

### 2.1 Literature search

We conducted a thorough systematic review and a comprehensive meta-analysis, primary on key outcomes, in alignment with the PRISMA 2020 criteria (23), adhering to the AMSTAR guidelines ensured the quality of assessment. Our systematic review is registered on the PROSPERO (registration number: [CRD42024578763]).

Two researchers independently conducted the literature search and study screening. In cases of disagreement, a third reviewer was consulted to make the final decision. Four databases were searched: Embase, PubMed, Cochrane Library, and Web of Science. The search period was from the inception of each database to August 2024. Search terms: PubMed (“Simplified PADUA”[Title/Abstract] OR SPARE[Title/Abstract] OR “Simplified PADUA Renal”[Title/Abstract] OR “SPARE nephrometry”[Title/Abstract] OR “Simplified PADUA REnal”[Title/Abstract]) AND(“Partial Nephrectomy”[Mesh] OR “Nephrectomy, Partial”Title/Abstract] OR “partial nephrectomy”[Title/Abstract] OR PN[Title/Abstract] OR “nephron-sparing surgery”[Title/Abstract] OR NSS[Title/Abstract]); Embase (‘simplified padua’:ti,ab,kw OR spare:ti,ab,kw OR ‘simplified padua renal’:ti,ab,kw OR ‘spare nephrometry’:ti,ab,kw OR ‘simplified padua renal nephrometry system’:ti,ab,kw) AND (‘partial nephrectomy’/exp OR ‘partial nephrectomy’:ti,ab,kw OR ‘nephron sparing surgery’:ti,ab,kw OR pn:ti,ab,kw OR nss:ti,ab,kw) Cochrane Library (“Simplified PADUA”:ti,ab,kw OR SPARE:ti,ab,kw OR “Simplified PADUA Renal”:ti,ab,kw OR “SPARE nephrometry”:ti,ab,kw OR “Simplified PADUA REnal”:ti,ab,kw) AND (MeSH descriptor: Nephrectomy, Partial] explode all trees OR “partial nephrectomy”:ti,ab,kw OR “nephron-sparing surgery”:ti,ab,kw OR PN:ti,ab,kw OR NSS:ti,ab,kw); Web of Science TS=((“Simplified PADUA” OR SPARE OR “Simplified PADUA Renal” OR “SPARE nephrometry” OR “Simplified PADUA REnal”) AND (“partial nephrectomy” OR “nephron-sparing surgery” OR PN OR NSS)). The literature search was restricted to studies published in English. In addition, the reference lists of relevant studies were manually screened to identify potentially eligible articles.

### 2.2 Eligibility criteria

Studies were included in this systematic review if they met the following criteria: (1) involved patients undergoing partial nephrectomy (PN); (2) assessed renal complexity using the SPARE scoring system; and (3) reported at least one perioperative outcome, such as operative time (OT), length of stay (LOS), estimated blood loss (EBL), change in estimated glomerular filtration rate (eGFR), warm ischemia time (WIT), or postoperative complications. Studies were excluded if they met any of the following criteria: (1) usable data could not be extracted; (2) they were editorials, conference abstracts, or expert opinions; (3) they were duplicate publications reporting identical findings in the same study population; (4) they involved non-human subjects; or (5) the SPARE scoring system was not used to assess the complexity or risk of PN.

### 2.3 Data extraction

Two independent reviewers selected eligible articles and extracted data using a standardized data collection form The extracted data included the following variables: author, year of publication, document type, sample size, age, body mass index (BMI), tumor size, surgical approach, operative time (OT), length of stay (LOS), estimated blood loss (EBL), warm ischemia time (WIT), change in estimated glomerular filtration rate (eGFR), TRIFECTA achievement, and PENTAFECTA achievement. Postoperative complications were extracted from each study. Complication severity was graded according to the Clavien–Dindo classification, and major complications were defined as Clavien–Dindo grade ≥3.

### 2.4 Study quality assessment

Retrospective studies were assessed using the Newcastle–Ottawa Scale (NOS) (24). NOS scores range from 0 to 9, with scores greater than 6 considered indicative of high methodological quality.

### 2.5 Risk of bias assessment

The two reviewers independently assessed the risk of bias in the included studies using the ROBINS-I tool, which is specifically designed for non-randomized studies. This tool evaluates bias across seven domains: bias due to confounding, bias in the selection of participants, bias in the classification of interventions, bias due to deviations from intended interventions, bias due to missing data, bias in outcome measurement, and bias in the selection of the reported result (25).

### 2.6 Data analysis

Statistical analyses were performed using Stata version 16.0 (StataCorp LLC, College Station, TX, USA). In this meta-analysis, log odds ratios (log ORs) and weighted mean differences (WMDs) were used to synthesize the results of the included studies (26). A two-sided P value < 0.05 was considered statistically significant. Heterogeneity among the included studies was assessed using the chi-square test and the Q test. Significant heterogeneity was defined as I² > 50% or P < 0.10, in which case a random-effects model was used. Subgroup analyses according to surgical approach were planned when sufficient data were available. However, because most included studies predominantly involved robot-assisted partial nephrectomy (RAPN), and data for open partial nephrectomy (OPN) or laparoscopic partial nephrectomy (LPN) were not separately or adequately reported, only limited RAPN subgroup analyses could be performed for selected outcomes.

## 3 Results

### 3.1 Description of study

**Figure 1:**
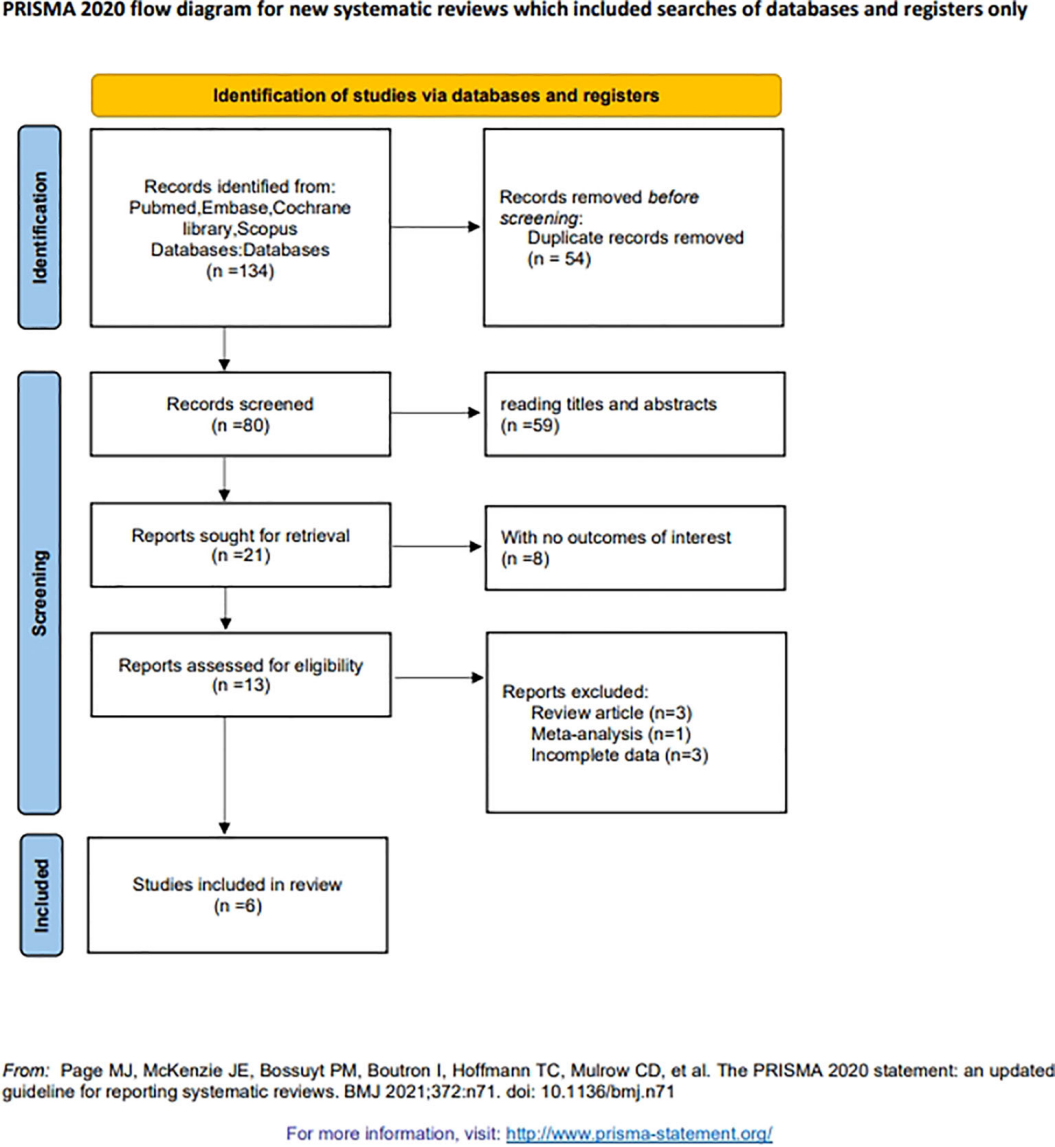
Flow diagram of the studies selection process.

A total of 134 records were identified through searches of four databases. After removal of 54 duplicate records, 97 studies were excluded after title and abstract screening. During full-text review, eight studies were excluded because they did not report outcomes of interest, three were systematic reviews, one was a meta-analysis, and three had incomplete data. Ultimately, six studies involving 1,831 patients were included in the meta-analysis. The sample size of the included studies ranged from 201 to 531 patients. All six included studies were retrospective in design (22, 27–31). The study selection process is presented in **Figure 1**, and the baseline characteristics of the included studies are summarized in **Table 1**. The six included studies were published between 2019 and 2024.

**Table 1:** Baseline data for studies included in the meta-analysis.

Author	Year	Type	Sample (n)	Age	Tumor size(cm)	Operation	BMI^a^ (kg/m2)	Center
Giuseppe Rosiello (27)	2022	Retrospective	368	64	3.2	RAPN^d^	27	Single-center
Gopal Sharma (29)	2022	Retrospective	201	52	4.2	RAPN	25.3	Single-center
Chi-Ping Huang (30)	2020	Retrospective	207	58	3.5	OPN^b^ LPN^c^ RAPN	25.6	Single-center
Samuel Weprin (31)	2021	Retrospective	202	57.5	2.3	RAPN	29.6	Single-center
Vincenzo Ficarra (22)	2019	Retrospective	531	64	3.2	OPN LPN RAPN	25.7	Multicenter study
Matthew G Crockett (28)	2021	Retrospective	322	62	3	RAPN	27.8	Single-center

BMI^a^, body mass index; OPN^b^, open partial nephrectomy; LPN^c^, laparoscopic partial nephrectomy; RAPN^d^, robot-assisted laparoscopic partial nephrectomy.

### 3.2 Quality assessment

The quality of the included cohort studies was assessed using the modified Newcastle–Ottawa Scale (NOS), with scores ranging from 6 to 7. All six studies were included in the quality assessment, and each received an NOS score of 6 or higher, as shown in **Table 2**.

**Table 2:** Quality score of included studies based on the NOS scale.

Study	Selection			Comparability			Exposure			Total stars
	^a^REC	^b^SNEC	^c^AE	^d^DO	^e^SC	^f^AF	^g^AO	^h^FU	^i^AFU	
Giuseppe Rosiello (27)	1	1	1		1	1	1	1		7
Gopal Sharma (29)	1	1	1	1	1		1		1	7
Chi-Ping Huang (30)	1	1	1	1				1	1	6
Samuel Weprin (31)	1	1	1	1	1	1	1			7
Vincenzo Ficarra (22)	1	1	1	1		1	1	1		7
Matthew G Crockett (28)	1	1	1		1		1		1	6

^a^ REC, representativeness of the cohort;

^b^ SNEC, selection of the none posed cohort;

^c^ AE, ascertainment of exposure;

^d^ DO, demonstration that outcome of interest was not present at start of study;

^e^ SC, study controls most important factors;

^f^ AF, study controls for other important factors;

^g^ AO, assessment of outcome;

^h^ FU, follow-up long enough for outcomes to occur;

^i^ AFU, adequacy of follow-up of cohort (≥ 80%).

### 3.3 Operation time

**Figure 2:**
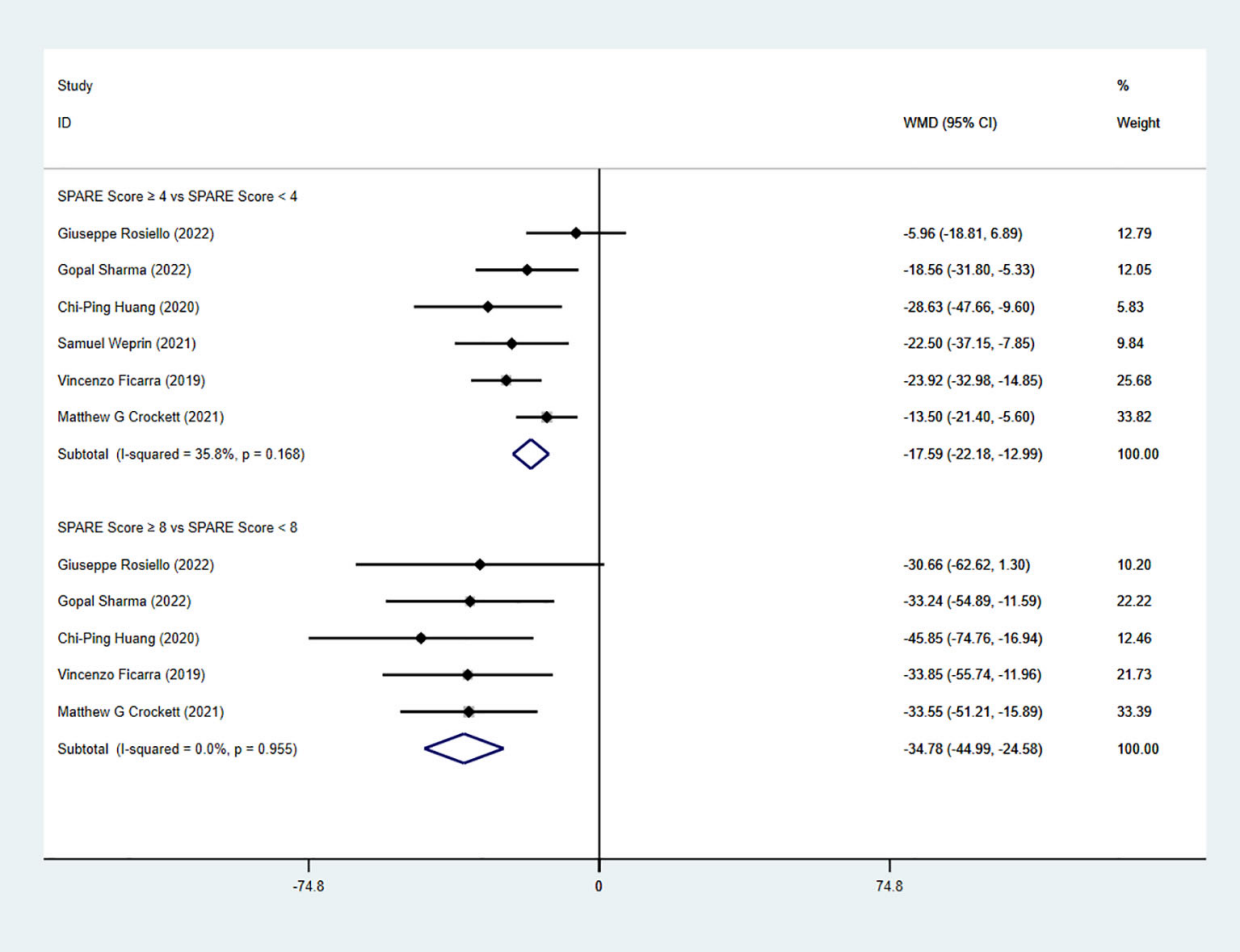
Forest plot and meta-analysis of OT.

**Figure 3:**
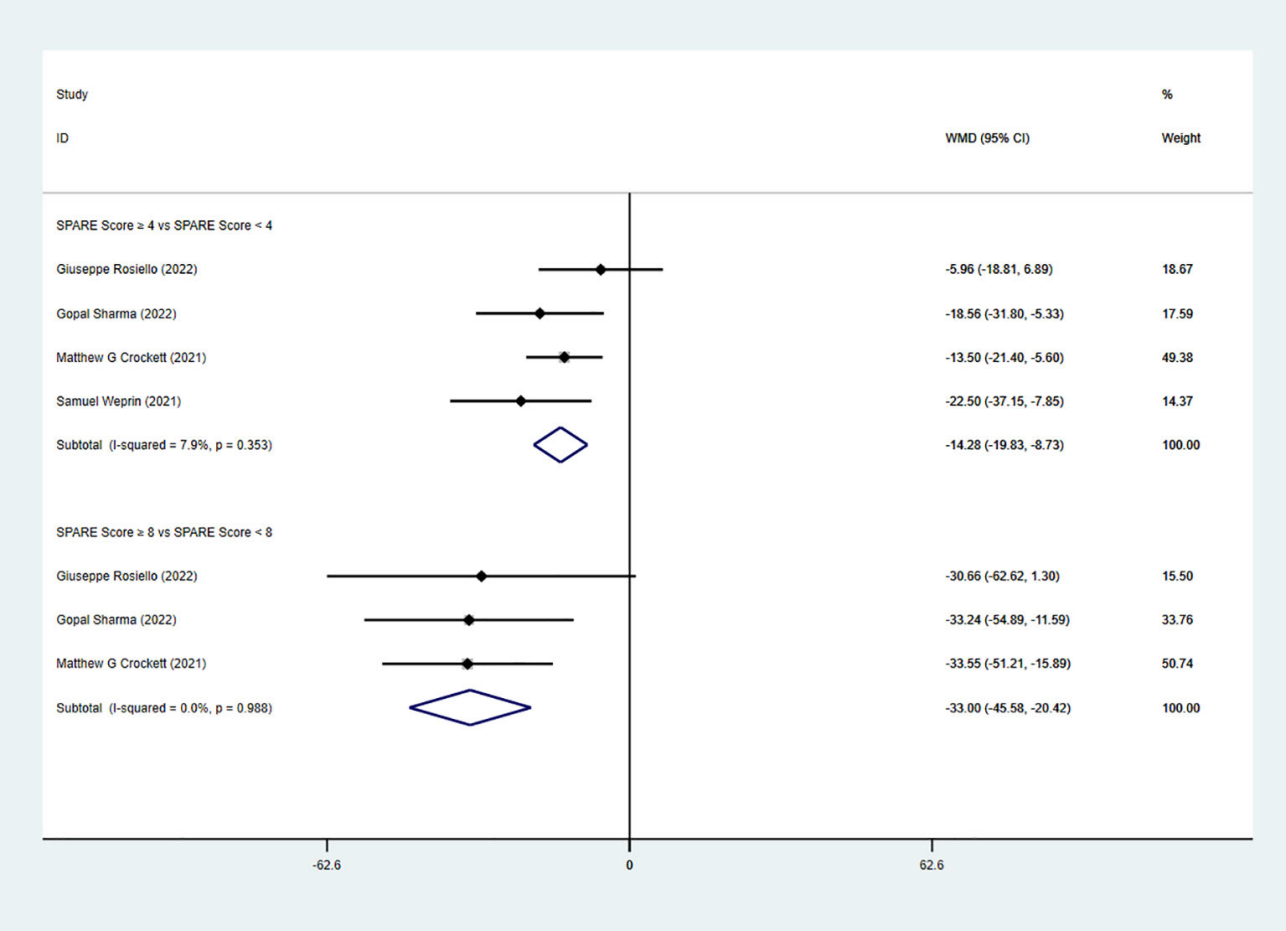
OT subgroup analysis with RAPN.

The pooled analysis showed a significant difference in operation time (OT) between the low-risk group (SPARE score 0–3) and the intermediate-risk group (SPARE score 4–7) (WMD = −17.59, 95% CI: −22.18 to −12.99, P < 0.05; I² < 50%; fixed-effects model). A significant difference in OT was also observed between the intermediate-risk group and the high-risk group (SPARE score 8–10) (five studies; WMD = −34.78, 95% CI: −44.99 to −24.58, P < 0.05; I² < 50%; fixed-effects model) (**Figure 2**). Subgroup analysis of RAPN also demonstrated significant differences in OT between the low-risk group and the intermediate-risk group (four studies; WMD = −14.28, 95% CI: −19.83 to -8.73, P < 0.05; I² < 50%; fixed-effects model), as well as between the intermediate-risk group and the high-risk group (3 studies; WMD = −33.00, 95% CI: −45.58 to −20.42, P < 0.05; I² < 50%; fixed-effects model) (**Figure 3**).

### 3.4 Length of stay

**Figure 4:**
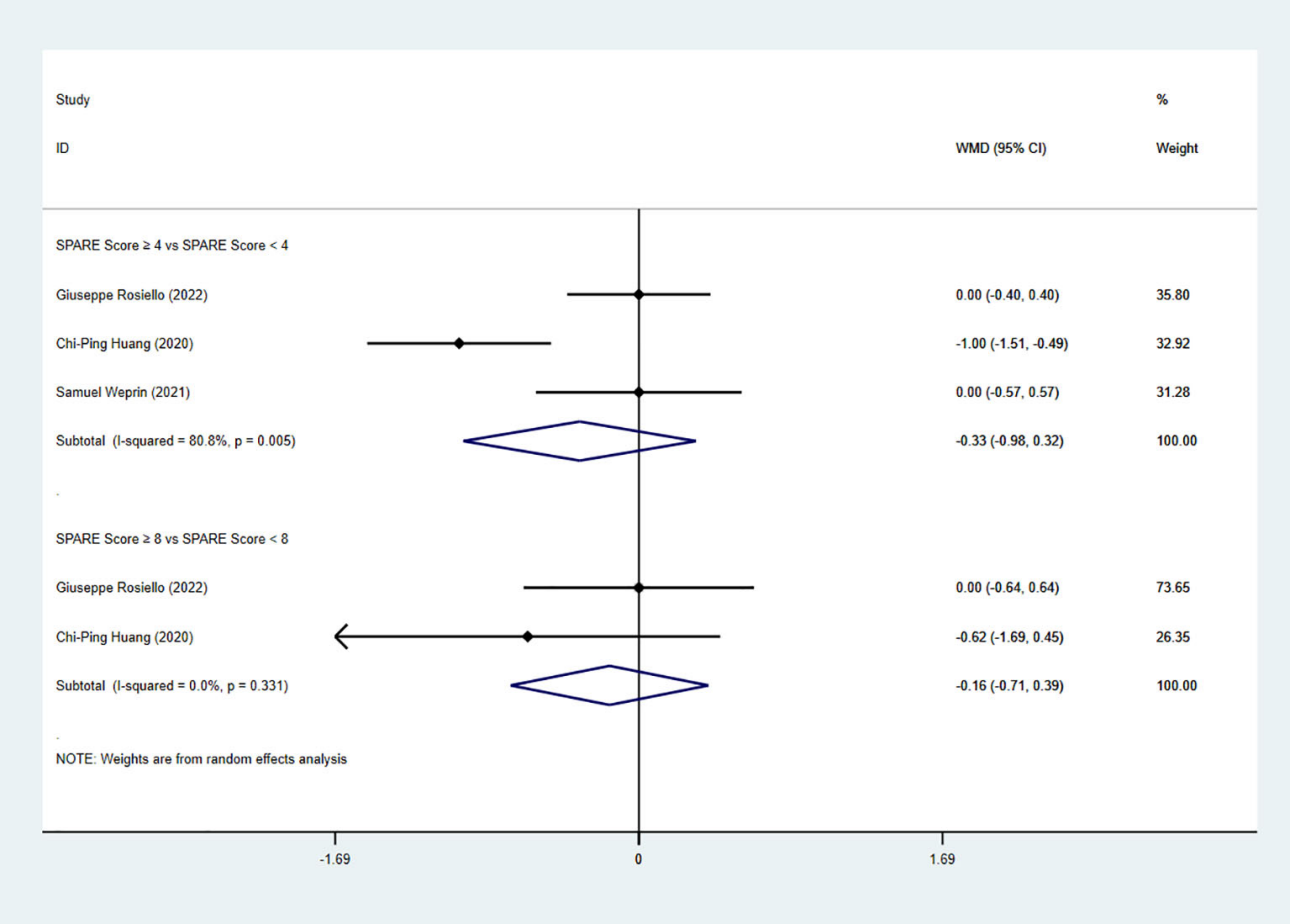
Forest plot and meta-analysis of LOS.

Three studies reported length of stay (LOS). The pooled analysis showed no significant difference between the low-risk group and the intermediate-risk group (WMD = −0.33, 95% CI: −0.98 to 0.32, P=0.32; I² ≥ 50%; random-effects model). However, given the high heterogeneity, likely stemming from varying discharge protocols, postoperative care pathways, and institutional practices, this pooled estimate should be interpreted with caution. Similarly, no significant difference was observed between the intermediate-risk group and the high-risk group (two studies; WMD = −0.16, 95% CI: −0.71 to 0.39, P=0.56; I² < 50%; fixed-effects model) (**Figure 4**).

### 3.5 Estimated blood loss

**Figure 5:**
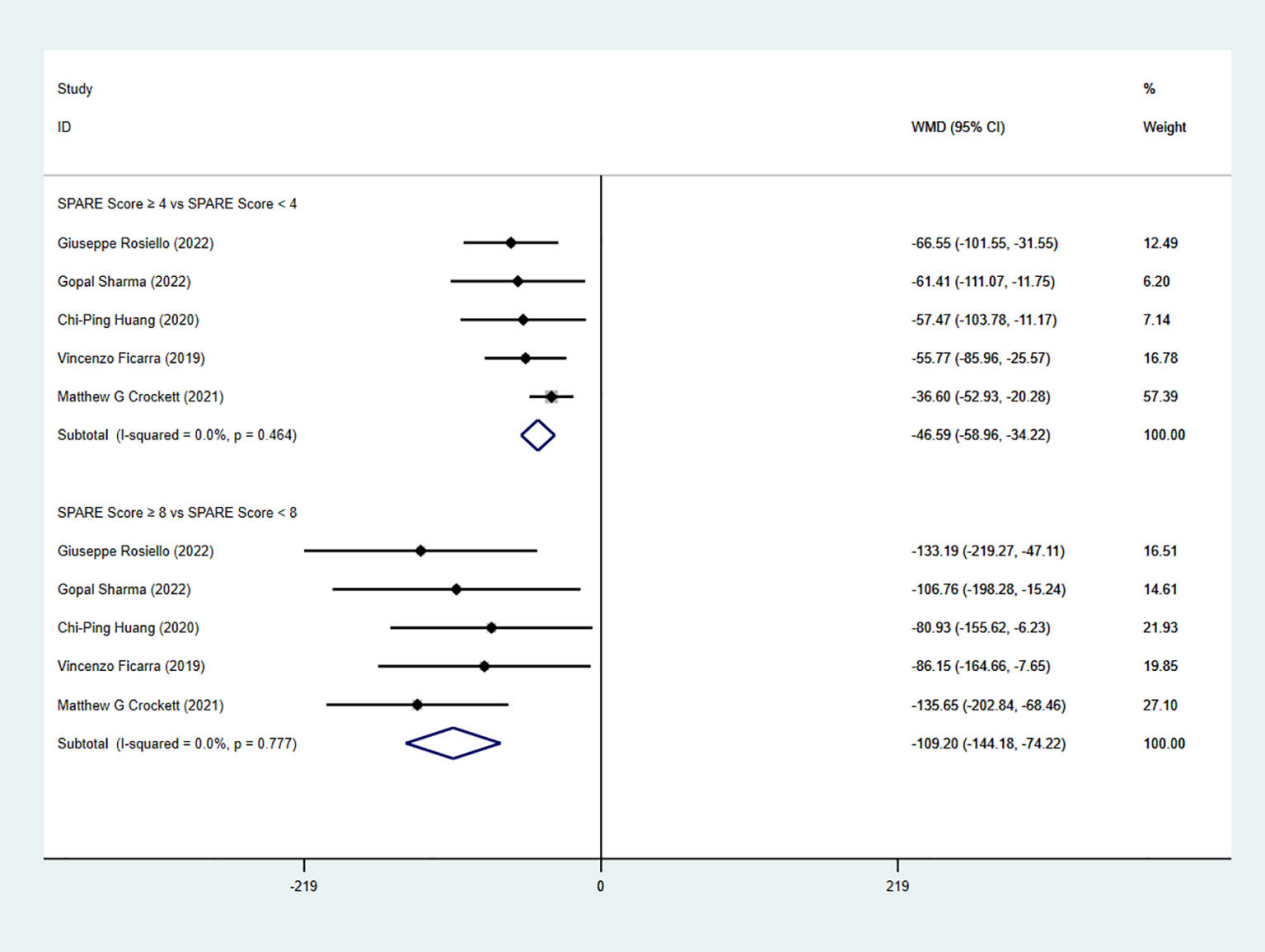
Forest plot and meta-analysis of EBL.

Five studies reported estimated blood loss (EBL). The pooled analysis demonstrated a significant difference between the low-risk group and the intermediate-risk group (WMD = −46.59, 95% CI: −58.96 to −34.22, P < 0.05; I² < 50%; fixed-effects model). A significant difference was also observed between the intermediate-risk group and the high-risk group (five studies; WMD = −109.20, 95% CI: −144.18 to −74.22, P < 0.05; I² < 50%; fixed-effects model) (**Figure 5**).

### 3.6 Warm ischemia time

**Figure 6:**
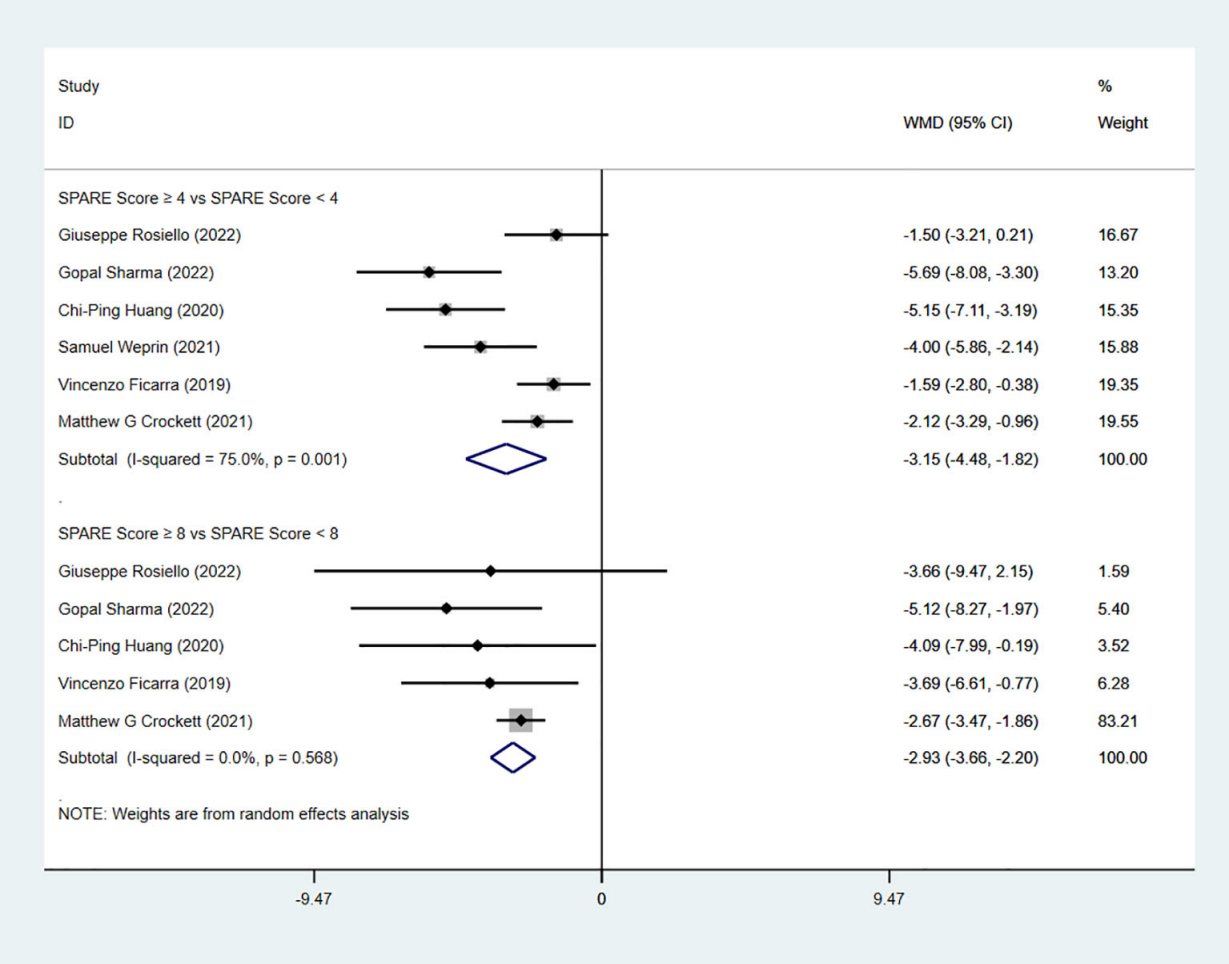
Forest plot and meta-analysis of WIT.

**Figure 7:**
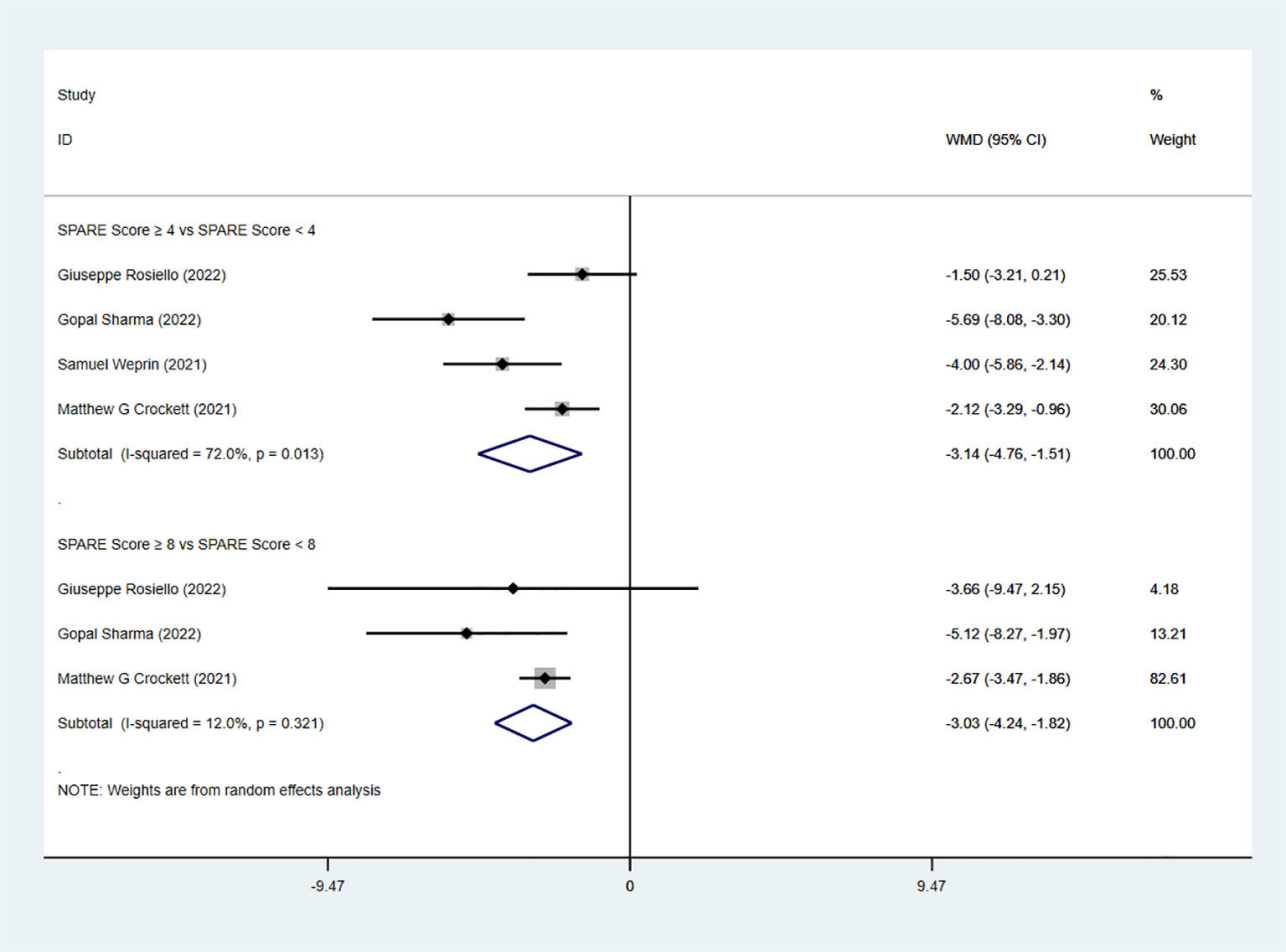
WIT subgroup analysis with RAPN.

Six studies reported warm ischemia time (WIT). The pooled analysis showed a significant difference between the low-risk group and the intermediate-risk group (WMD = −3.15, 95% CI: −4.48 to −1.82, P < 0.05; I² ≥ 50%; random-effects model). A significant difference was also observed between the intermediate-risk group and the high-risk group (five studies; WMD = −2.93, 95% CI: −3.66 to −2.20, P < 0.05; I² < 50%; fixed-effects model) (**Figure 6**). In the RAPN subgroup analysis, significant differences in WIT were observed both between the low-risk group and the intermediate-risk group (four studies; WMD = −3.14, 95% CI: −4.76 to −1.51, P < 0.05; I² ≥ 50%; random-effects model) and between the intermediate-risk group and the high-risk group (three studies; WMD = −3.03, 95% CI: −4.24 to −1.82, P < 0.05; I² < 50%; fixed-effects model) (**Figure 7**).

### 3.7 eGFR change

**Figure 8:**
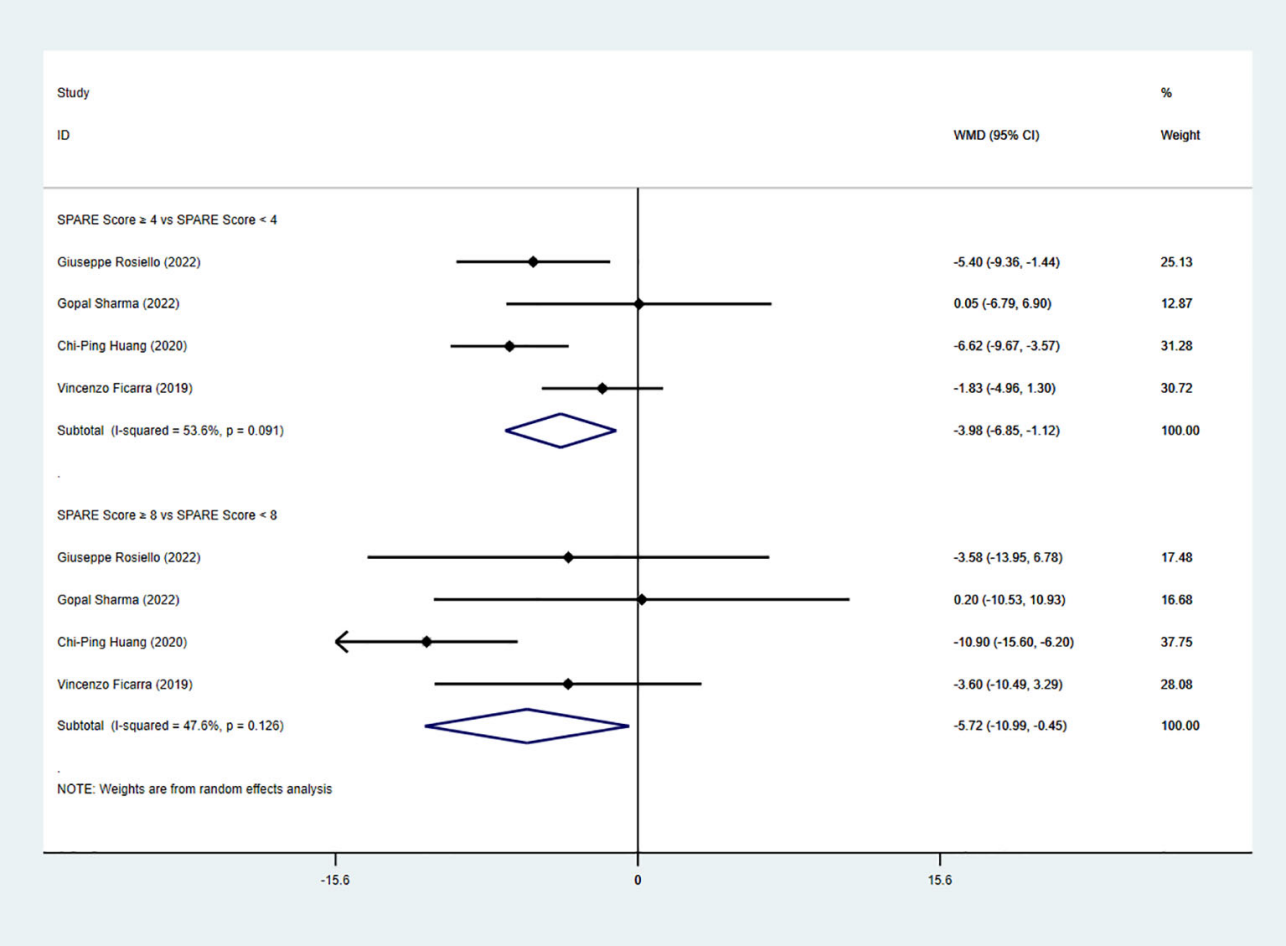
Forest plot and meta-analysis of eGFR change.

Four studies reported change in estimated glomerular filtration rate (eGFR). The pooled analysis demonstrated a significant difference between the low-risk group and the intermediate-risk group (WMD = −3.98, 95% CI: −6.85 to −1.12, P < 0.05; I² ≥ 50%; random-effects model). A significant difference was also observed between the intermediate-risk group and the high-risk group (four studies; WMD = −5.72, 95% CI: −10.99 to −0.45, P < 0.05; I² < 50%; fixed-effects model) (**Figure 8**).

### 3.8 Complications

**Figure 9:**
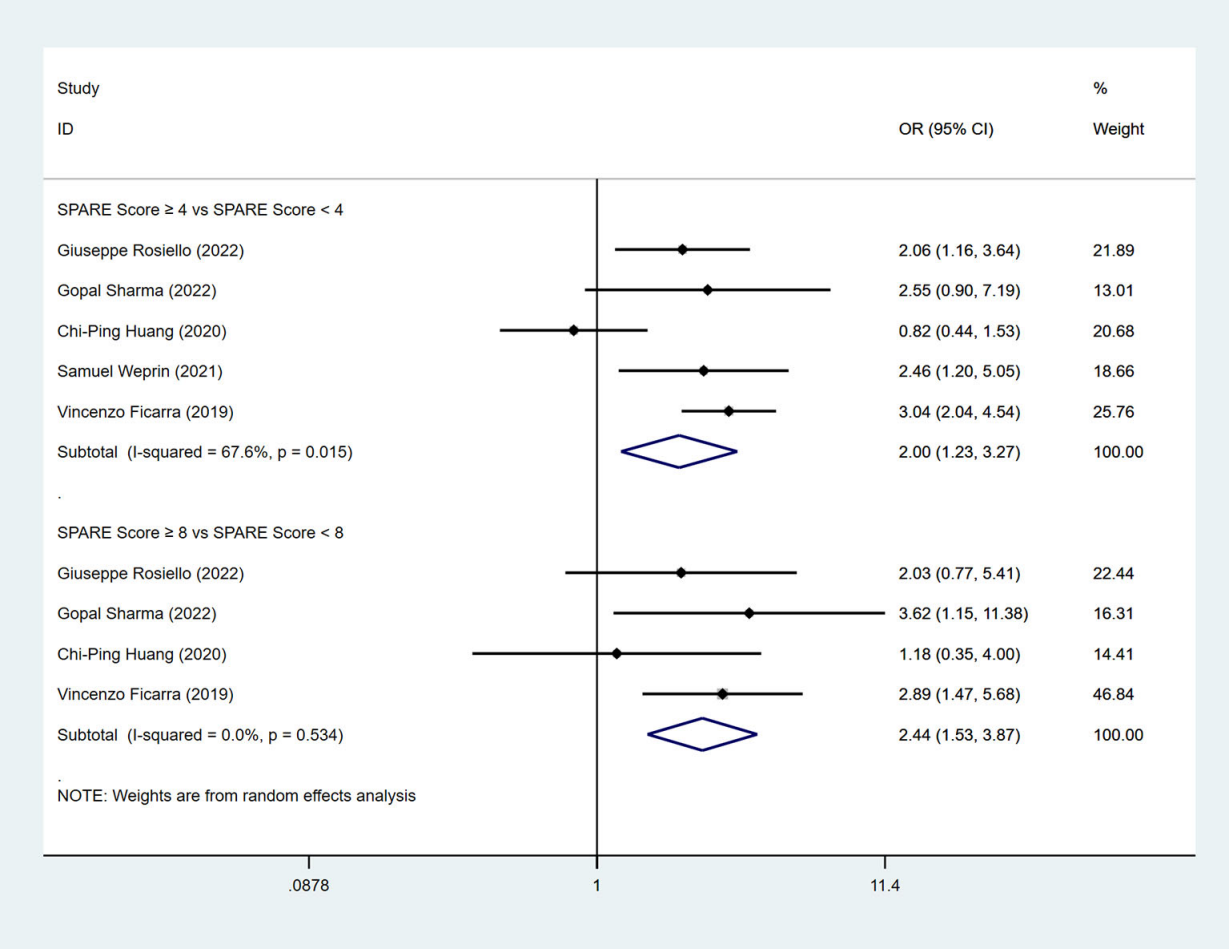
Forest plot and meta-analysis of complications.

Five studies reported postoperative complications. The pooled analysis showed a significant difference between the low-risk group and the intermediate-risk group (OR=2.00, 95% CI: 1.23 to 3.27, P < 0.05; I² ≥ 50%; random-effects model). A significant difference was also observed between the intermediate-risk group and the high-risk group (four studies; OR=2.44, 95% CI: 1.53 to 3.87, P < 0.05; I² < 50%; fixed-effects model) (**Figure 9**).

### 3.9 TRIFECTA achievement

**Figure 10:**
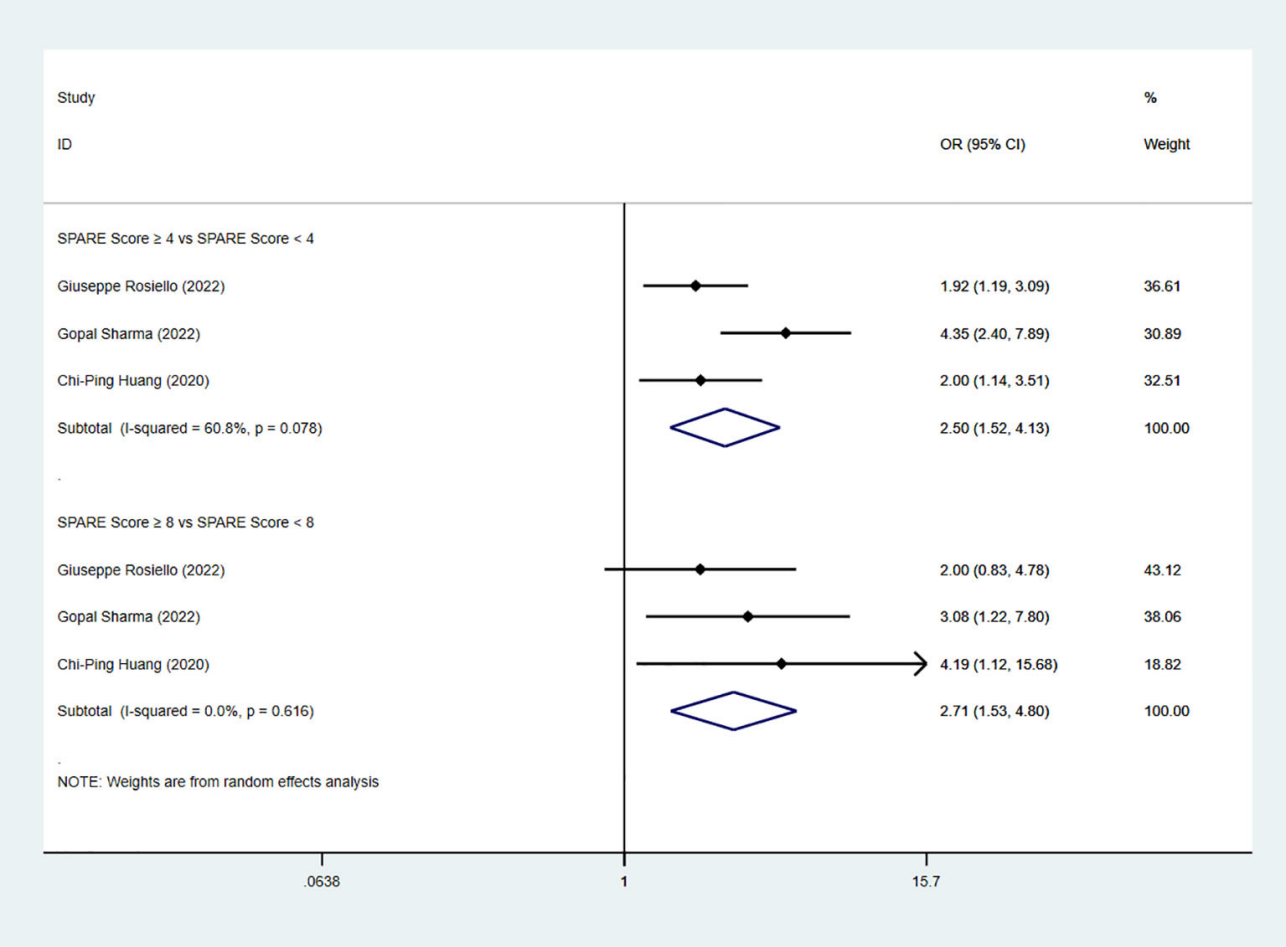
Forest plot and meta-analysis of TRIFECTA achievement.

Three studies reported TRIFECTA achievement. The pooled analysis demonstrated a significant difference between the low-risk group and the intermediate-risk group (OR=2.50, 95% CI: 1.52 to 4.13, P < 0.05; I² ≥ 50%; random-effects model). A significant difference was also observed between the intermediate-risk group and the high-risk group (three studies; OR=2.71, 95% CI: 1.53 to 4.80, P < 0.05; I² < 50%; fixed-effects model) (**Figure 10**).

### 3.10 PENTAFECTA achievement

**Figure 11:**
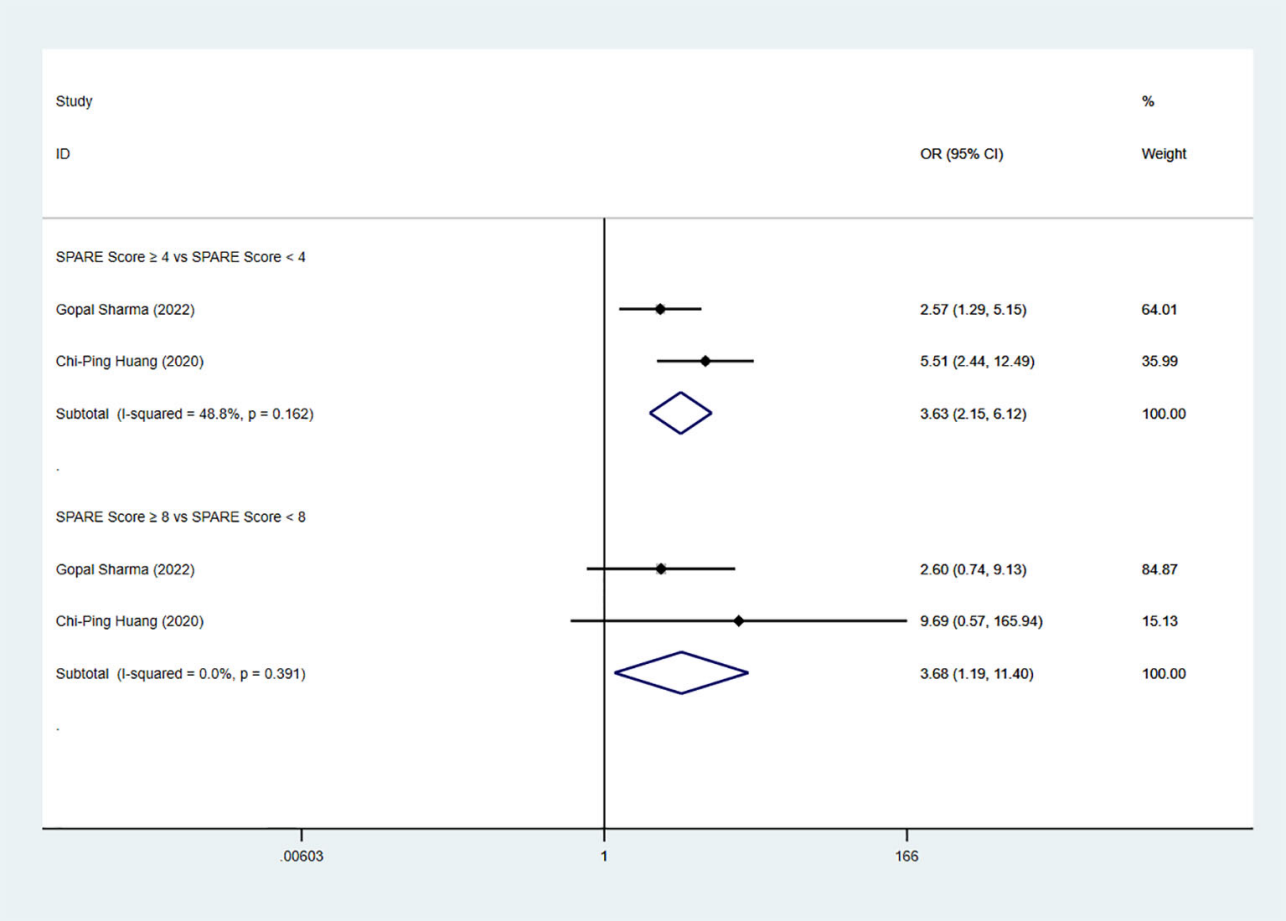
Forest plot and meta-analysis of PENTAFECTA achievement.

Two studies reported PENTAFECTA achievement. The pooled analysis showed a significant difference between the low-risk group and the intermediate-risk group (OR=3.63, 95% CI: 2.15 to 6.12, P < 0.05; I² < 50%; fixed-effects model). A significant difference was also observed between the intermediate-risk group and the high-risk group (two studies; OR=3.68, 95% CI: 1.19 to 11.40, P < 0.05; I² < 50%; fixed-effects model) (**Figure 11**).

### 3.11 Sensitivity analysis

Sensitivity analyses were performed to assess the robustness of the pooled results and to explore the influence of individual studies on heterogeneity for each outcome. Overall, the direction and significance of the pooled estimates remained stable after sequential exclusion of individual studies, suggesting that no single study disproportionately influenced the results. However, for outcomes with substantial heterogeneity, including LOS, WIT, complications, TRIFECTA achievement, and eGFR change, the observed variability was likely attributable not only to statistical fluctuation but also to differences in study design, surgical approach, baseline patient characteristics, perioperative management, and outcome definitions across the included studies. Because only a limited number of studies were available for these outcomes, further meta-regression or more detailed subgroup analyses were not feasible.

### 3.12 Publication bias

Formal assessment of publication bias using funnel plots or Egger’s regression test was not performed because each pooled analysis included fewer than 10 studies. Under these conditions, these methods are generally considered unreliable; therefore, the results were not interpreted. Nevertheless, the possibility of publication bias cannot be excluded.

## 4 Discussion

The present meta-analysis suggests that higher SPARE scores are associated with less favorable perioperative outcomes after partial nephrectomy, including longer operative time, greater estimated blood loss, prolonged warm ischemia time, greater postoperative renal functional decline, a higher risk of complications, and lower rates of TRIFECTA and PENTAFECTA achievement.

The pooled results showed that operative time was longer in patients with higher SPARE scores. This finding is clinically plausible, as greater tumor complexity—particularly with respect to tumor size and its relationship to adjacent structures—often necessitates more technically demanding procedures to achieve complete tumor excision while preserving surrounding tissues. In addition, the need to manage complex intraoperative situations may further prolong the duration of surgery (32, 33). These findings are consistent with the reports of Pettus et al. (34) and Potretzke et al. (35) which showed that larger tumor diameter and unfavorable tumor location increase the technical difficulty of PN and are associated with longer operative time. However, no significant difference was observed for LOS, and substantial heterogeneity was present across studies. LOS is strongly influenced by institutional discharge criteria, postoperative care pathways, and healthcare resource allocation, which may differ considerably among centers. Variation in surgical approach, surgeon experience, and perioperative recovery protocols may also affect the duration of hospitalization independently of tumor complexity. Differences in patient comorbidities and postoperative complication profiles may further contribute to variability in LOS across studies. With regard to estimated blood loss (EBL), higher SPARE scores were associated with greater intraoperative blood loss. Patients in the high-SPARE-score group are more likely to present with larger tumors and more anatomically complex lesions. These features often necessitate more extensive tissue dissection and resection of a larger volume of renal parenchyma (36). A previous meta-analysis also showed that host factors, particularly tumor size and complexity, influence perioperative outcomes after PN (37). During tumor excision, surgeons may require additional time to ensure complete resection and maintain hemostasis, which may consequently prolong warm ischemia time (WIT). Mottrie et al. (38) and Springer et al. (39) identified tumor location and complexity as critical determinants of WIT, both of which are closely reflected by the SPARE score. Prolonged WIT and a greater volume of renal parenchymal resection may contribute to ischemic injury and subsequent postoperative renal functional decline.

Higher SPARE scores were also associated with decline in eGFR. Mechanistically, this finding is plausible because more complex tumors often require longer warm ischemia time and may necessitate a greater volume of renal parenchymal resection, both of which can contribute to postoperative renal functional loss. However, the absolute pooled differences in eGFR were modest, and their clinical significance should be interpreted cautiously. In patients with preserved preoperative renal function, a mean decline of approximately 4–6 mL/min/1.73 m² may have limited short-term clinical impact. In contrast, in patients with impaired baseline renal reserve, preexisting chronic kidney disease, a solitary kidney, or limited contralateral renal compensation, even a modest additional decline may be clinically meaningful and may increase the risk of chronic kidney disease progression. It is also important to distinguish short-term from long-term renal functional outcomes. Prior evidence indicates that kidney function often worsens in the immediate postoperative period and then partially recovers over the following months before reaching a relatively stable level. Therefore, pooled postoperative eGFR change may reflect early functional injury rather than durable long-term renal impairment alone. In the studies by Rosiello et al. (27) and Huang et al. (30), a high SPARE score was associated with stage progression of chronic kidney disease and postoperative acute kidney injury, suggesting that tumor complexity may still have clinically relevant implications in selected patients. Nevertheless, the long-term impact of SPARE-related complexity on renal function remains influenced by follow-up duration, baseline renal function, contralateral kidney compensation, and perioperative renal-protection strategies. The heterogeneity observed for eGFR change likely reflects multiple sources. These may include differences in baseline renal function, contralateral kidney compensation, tumor size and location, extent of preserved renal parenchyma, ischemia technique and duration, use of renal-protection strategies, and variation in the timing and method of postoperative eGFR assessment among studies. The incidence of complications is one of the key indicators used to evaluate surgical outcomes. As the SPARE score increased, the incidence of complications also increased, including intraoperative bleeding, urine leakage, and renal functional impairment. Previous studies (40–43) have suggested that surgery is relatively less complex for tumors located on the lateral aspect of the kidney. In contrast, medially located tumors or those involving the renal sinus may require more meticulous surgical manipulation because of their close proximity to major vessels and the collecting system. The exophytic rate describes the extent to which the tumor protrudes from the renal surface. A higher exophytic rate is generally associated with less involvement of critical renal structures and, therefore, lower surgical complexity. Conversely, tumors with a higher endophytic component may be more closely associated with major vessels and the collecting system, thereby increasing surgical difficulty. Consequently, the risk of intraoperative and postoperative complications may be increased. TRIFECTA achievement is widely used as a composite measure for evaluating outcomes after PN. It includes three criteria: negative surgical margins, WIT <25 min, and the absence of major complications (44). Tumor size and surgical approach have both been associated with TRIFECTA achievement (44, 45). In the setting of complex renal tumors, difficulty in achieving TRIFECTA is mainly related to prolonged operative time and an increased risk of complications. PENTAFECTA achievement provides a more comprehensive assessment of surgical outcomes. In addition to the three TRIFECTA criteria, it includes preservation of more than 90% of estimated glomerular filtration rate (eGFR) and no upgrading of chronic kidney disease stage at 12 months. For tumors with greater anatomical complexity, PENTAFECTA is generally more difficult to achieve (46).

High heterogeneity was observed in WIT, complications, and TRIFECTA; the heterogeneity observed in WIT may also be explained by differences in surgical approach, hilar clamping strategy, selective versus main-artery ischemia, renorrhaphy technique, surgeon experience, and institutional operative preferences. Similarly, heterogeneity in complication outcomes may reflect variation in patient comorbidity profiles, surgical approach, surgeon expertise, perioperative care pathways, and inconsistent definitions or grading of complications across studies, particularly with respect to the inclusion of minor versus major complications. Furthermore, heterogeneity in TRIFECTA outcomes may be related to the composite nature of this endpoint, because it is influenced simultaneously by ischemia management, complication reporting, surgical expertise, and possible differences in the exact criteria or reporting standards used across studies. Because TRIFECTA and PENTAFECTA incorporates WIT and major complications, which were also analyzed separately, its association with SPARE score should be interpreted as partially overlapping with these component outcomes rather than as fully independent evidence.

Compared with other nephrometry systems used in partial nephrectomy, SPARE appears to offer several practical advantages, but also some limitations. Traditional anatomy-based systems such as PADUA and R.E.N.A.L. include a greater number of anatomical descriptors and have been widely used for preoperative risk stratification (47). By contrast, SPARE was developed as a simplified version of PADUA that retains several key tumor characteristics, including rim location, renal sinus involvement, exophytic rate, and tumor size, while reducing the number of variables required for scoring (48). From a clinical perspective, this simplification may facilitate routine preoperative application, reduce scoring burden, and potentially improve interobserver reproducibility. These features are particularly relevant in daily practice, where a practical and easily reproducible tool may be preferable to a more complex system (49). However, the simplified structure of SPARE may also come at the cost of reduced anatomical granularity compared with more detailed systems such as PADUA or R.E.N.A.L., and it does not capture all factors that may influence surgical difficulty, such as perinephric fat characteristics, surgeon experience, or institutional technique (50). In addition, although previous comparative studies have evaluated SPARE alongside PADUA and R.E.N.A.L., the currently available evidence remains insufficient to conclude that SPARE is definitively superior to other nephrometry systems across all perioperative endpoints. Therefore, SPARE may be best regarded as a practical and clinically useful alternative rather than a universal replacement for existing scoring systems.

An important limitation is the risk of residual confounding and selection bias inherent to the included studies, all of which were retrospective in design. Although higher SPARE scores were associated with worse perioperative outcomes, these relationships may have been influenced by factors not uniformly adjusted for across studies. In particular, tumor size, tumor location, baseline renal function, comorbidity burden, surgeon experience, surgical approach, clamping strategy, renorrhaphy technique, and institutional case volume may independently affect operative time, blood loss, warm ischemia time, postoperative complications, and renal functional outcomes. Moreover, treatment allocation in retrospective cohorts is not random, and patients with more complex tumors may have been preferentially managed by specific surgeons, centers, or operative approaches, thereby introducing selection bias. Because these confounders were not consistently reported or controlled for in the original studies, the pooled findings should be interpreted as associative rather than strictly causal.

Our study has several limitations. First, the number of available publications was limited, and unpublished studies were not accessible, which may have introduced publication bias. Second, the limited number of studies available for subgroup analysis made it difficult to accurately assess perioperative outcomes across different SPARE score categories. In addition, only a subset of perioperative outcomes could be analyzed in the RAPN subgroup. Thirdly, substantial heterogeneity was observed in several outcomes, including LOS, WIT, eGFR change, complications, and TRIFECTA achievement. Although sensitivity analyses suggested that the overall findings were relatively robust, the limited number of included studies prevented formal meta-regression or broader subgroup analyses to further identify the sources of heterogeneity. Therefore, these pooled estimates should be interpreted cautiously. TRIFECTA and PENTAFECTA were pooled from only three and two studies, respectively, which limits the precision and robustness of these estimates. These results should therefore be interpreted as exploratory rather than definitive. In addition, interpretation of renal functional outcomes should be cautious because the observed pooled decline in eGFR was modest and may not have the same clinical significance across all patients; its impact is likely to be greater in those with reduced baseline renal reserve or preexisting chronic kidney disease. Another important limitation is the currency of the literature search. The systematic search was conducted up to August 1, 2024; therefore, studies published after this date were not included in the quantitative synthesis. Several recent validation and comparative studies of the SPARE nephrometry system have been published since the search cutoff, including studies evaluating SPARE in robot-assisted and single-port robot-assisted partial nephrectomy settings. The absence of these newer studies may affect the completeness and contemporary relevance of the evidence base, particularly regarding the comparative performance of SPARE versus PADUA, R.E.N.A.L., and other nephrometry systems. Therefore, the present findings should be interpreted as a synthesis of the evidence available up to August 2024, and future updated meta-analyses incorporating post-cutoff studies are warranted.

## 5 Conclusion

The SPARE score is significantly associated with several perioperative outcomes after partial nephrectomy, including operative time, estimated blood loss, warm ischemia time, postoperative renal functional change, complications, and TRIFECTA/PENTAFECTA achievement. These findings suggest that SPARE may serve as a practical tool for preoperative assessment of tumor complexity and perioperative risk stratification. In the RAPN subgroup, higher SPARE scores were also associated with longer operative time and warm ischemia time. However, given the observational nature of the included studies, these findings should be interpreted as associative rather than as evidence of independent predictive superiority.

## Data Availability

The original contributions presented in the study are included in the article/**supplementary material**. Further inquiries can be directed to the corresponding author.
