# Supplementary figures and images for: Simplified PADUA renal nephrometry system, an imaging features scoring system, predicts perioperative outcomes in partial nephrectomy: a meta-analysis
Source: Front Oncol. 2026 May 20;16:1684584. doi: 10.3389/fonc.2026.1684584 (PMC13229746; doi:10.3389/fonc.2026.1684584)

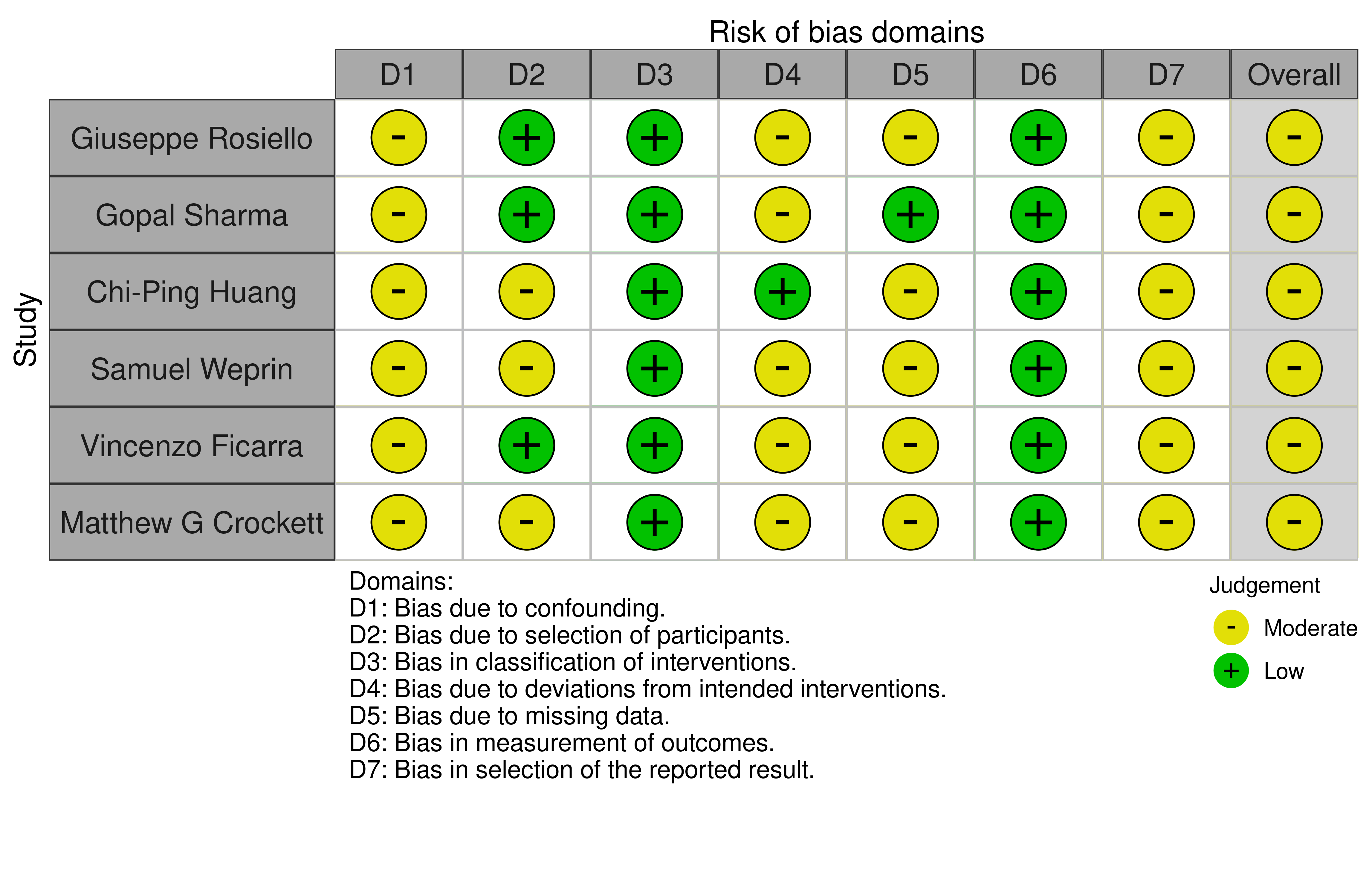

Supplement: Supplementary file 1 [file Image1.png]
